# Coping with Environmental Extremes: Population Ecology and Behavioural Adaptation of *Erebia pronoe*, an Alpine Butterfly Species

**DOI:** 10.3390/insects12100896

**Published:** 2021-10-01

**Authors:** Martin Wendt, Nele Senftleben, Patrick Gros, Thomas Schmitt

**Affiliations:** 1 Senckenberg German Entomological Institute, Systematics and Biogeography, Eberswalder Str. 90, 15374 Müncheberg, Germany; n.senftleben@gmail.com (N.S.); Thomas.Schmitt@senckenberg.de (T.S.); 2 Haus der Natur Salzburg, WorkGroup Entomology, 5020 Salzburg, Austria; patrick.gros@hausdernatur.at; 3 Zoology, Institute of Biology, Faculty of Natural Sciences I, Martin Luther University Halle-Wittenberg, 06099 Halle (Saale), Germany; 4 Entomology and Biogeography, Institute of Biochemistry and Biology, Faculty of Science, University of Potsdam, 14476 Potsdam, Germany

**Keywords:** mark-release-recapture, movement patterns, opportunistic behaviour, partial protandry, population demography

## Abstract

**Simple Summary:**

High alpine meadows are home to numerous endemic butterfly species. A combination of climate change and changes in agricultural practices has led to a severe decline in many species. A seemingly unaffected representative of this habitat is *Erebia pronoe*. We studied the behaviour, resource use and population structure of this species to explain its resilience and estimate its future survival potential. This species shows pronounced protandry in combination with serial eclosion. Males were significantly more active and mobile and were also caught significantly more often than females, resulting in a pronounced shift in sex ratio in the predicted population structure. The adults use a wide range of nectar plants and establish homeranges in areas of high habitat quality. Thus, *Erebia pronoe* adults use a wide array of resources combined with a slight specialisation to avoid niche overlap with closely related species. The resulting ecological flexibility seems to be an adaptation to unpredictable environmental conditions, which should be the result of a long-lasting adaptation process. Moreover, the combination of opportunism and modest specialisation should also be a good basis for coping with future changes caused by climate and land-use change.

**Abstract:**

A mark-recapture study of the nominotypical *Erebia pronoe* in the Alps was conducted to survey its ecological demands and characteristics. Population structure analysis revealed a combination of protandry (one-week earlier eclosion of males) and serial eclosion. Significant differences between both sexes were found in population density (males: 580/ha ± 37 SE; females: 241/ha ± 66 SE), sex-ratio (2.4) and behaviour (57.7 vs. 11.9% flying). Both sexes used a wide range of nectar plants (Asteraceae, 77.3%; Dipsacaceae, 12.3%; Gentianaceae, 9.7%). The use of nectar plants shows a non-specific spectrum, which, however, completely avoids overlap with the locally co-occurring species *Erebia nivalis*. Movement patterns show the establishment of homeranges, which significantly limits the migration potential. Due to its broad ecological niche, *E. pronoe* will probably be able to react plastically to the consequences of climate change. The formation of high population densities, the unconcerned endangerment status, the unspecific resource spectrum and the sedentary character of the species make *E. pronoe* a potential indicator of the quality and general resource occurrence of alpine rupicolous grasslands.

## 1. Introduction

The worldwide losses in species diversity and abundance are so strong that the recent extinction of species is compared with the major extinction events in earth’s history [1]. Although biodiversity loss is a global phenomenon, individual regions, habitats and species groups are particularly affected. For instance, as a result of their generally lower plasticity, specialised stenotopic species are assumed to be more vulnerable to biotic and abiotic disturbances, as they do not have the ecological flexibility to react plastically to the numerous biotic and abiotic disturbances [2]. Especially extreme habitats, with the alpine environments being one, require specific adaptations to the prevailing harsh conditions and are thus home to many specialised and narrow-ranging species [3]. For this reason, Europe’s high mountains are important centres of biodiversity [4].

Habitat loss is one of the main drivers of the current biodiversity crisis [5]. With the loss of high-quality habitats, low-mobility species are affected in particular, as dispersal and/or maintenance of metapopulations is only possible to a limited extent. This is especially true for montane and alpine species, whose extinction risk is further increased by increasing isolation [6] since their habitats are inherently more susceptible to fragmentation [7] and their populations are more limited due to species–arearelationship [8]. Additional pressure is caused by the ongoing climate change, which is causing range shifts in many species in order to maintain their ecological niches [9,10]. Nevertheless, it is not possible for numerous species to keep up with climate change, thus resulting in an extinction debt [2]. Another risk of climatic change is a potential inter-and intraspecific decoupling [11,12,13], which can negatively affect population size [14]. Similarly, climatic conditions affect the resource supply and quality of a habitat, which in turn affects the reproductive rate [15] and thus the population size of the species found there. The population structures of a species can strongly influence behaviour [16], and low abundance can lead to negative feedback [13]. Therefore, to determine the status of a habitat or a potential indicator species, an analysis of its population structure in combination with migration patterns, resource use and behaviour must be examined.

The sensitive reaction of insects to environmental changes and their high reproductive rate qualify them as excellent indicators and study objects for changes of habitat quality [2,17]. Especially, butterflies have become a preferred study group for analysing the quality and linkage of habitats due to their ecological specialisation and mobility [6,18,19].

We have selected the butterfly species *Erebia pronoe* as a characteristic species of the endangered flower-rich montane and alpine grasslands to evaluate its ecological needs, its potential as an indicator species and its future prospects. *Erebia pronoe* is an excellent representative of this habitat type because of its overall abundance and its wide distribution in the high mountains of Europe [20] but also in numerous prime butterfly areas [21]. In this paper, the population structure and demographic development of this montane butterfly were analysed based on a mark-release-recapture study in the Austrian Alps. The region harbours the nominotypical taxon *E. pronoe pronoe*. This study provides essential information about the specific adaptations to high mountain habitats, especially regarding the potential expression of protandric structures [22,23]. Additionally, the sex-specific mobility of individuals was investigated to derive conclusions about habitat linkage and metapopulation structure in the region under investigation. We were able to analyse the effect of climatic factors on the behaviour, the sex-specific behaviour and the resourceuse of *Erebia p. pronoe*. Thus, the following two key questions are the focus of our investigation:

Does *E. p. pronoe* have specific adaptations of its population demography (e.g., modifications in protandry, prolonged eclosion phases) that can be understood as specific adaptations to its habitats, and will these allow the species to adapt to expected future climatic changes?

Does *E. p. pronoe* exhibit sufficient ecological flexibility and mobility to be able to survive the expected further changes in its alpine environment?

## 2. Materials and Methods

### 2.1. Study Species

*Erebia pronoe* belongs to the western Palaearctic Nymphalidae. The species can be found on wet meadows, calcareous grasslands and screes from the high montane to the alpine zone [24]. *E. pronoe* is widespread and often common throughout the Alps. It is locally found in the Swiss and French Jura as well as in the Pyrenees, where high densities can be reached. In the Carpathians, including the High Tatras and the mountains of the Balkan Peninsula, populations of *E. pronoe* are relatively rare and small [25]. The species flies in one generation from the end of July to mid-September and hibernates as L1 larva. The larvae prefer Festucaspecies such as *F. ovina*, *F. rubra* and *F. quadriflora,* and to a lesser degree *Anthoxanthum odoratum*; breeding was successful on *Poa annua* [26].

### 2.2. Study Area

The study area is located in the Austrian Alps in the core of the distribution area of the nominal subspecies in the “Hohe Tauern National Park” near the “Haus der Alpinen Naturschau” (47°07′ N,12°49′ E), in the state of Salzburg. The investigated steep slope covers an area of 4.6 ha and extends at an altitude of 2250–2400m asl. The area is interspersed with gravel areas and represents a characteristic habitat of *E. pronoe*. Other suitable habitats are present within a distance of 200 m. To the north and east, it borders on the Special Protection Area “Piffkar-Fusch”, where no collection was allowed. The area in the west was too steep, and the “Untere Nassfeld” in the south was too heavily grazed for working. The flat areas at the foot of the slope were grazed by 19 cows.

### 2.3. Mark-Release-Recapture Study

We conducted a mark-release-recapture study as the basis for our analyses of population structure and species-specific mobility parameters. Furthermore, behaviour and specific resource use were surveyed to identify specific adaptations to alpine habitats. The study was carried out from 16 July 2019 to 1 September 2019, covering most of the flight season of *E. pronoe*. The butterflies were caught with a 40 cm diameter butterfly net under suitable conditions (see [27]) from 9 am to 6 pm; to avoid day-time effects, the sampling was always started at different sections. Each individual was marked on the underside of the hind wings with a fine, waterproof pen (StabiloOHPen universal S) with an individual code consisting of letters for the day and a consecutive number. In addition, GPS coordinates, sex, wing condition (scale 1 = wing seam completely preserved; up to 4 = wing heavily damaged [28]), behaviour when being captured and current weather conditions were recorded. The same data were collected for each recapture, excluding recaptures during the same day, to avoid behavioural effects of the capture and theoretically allow complete mixing of the population [29]. We assessed the age structure of the population based on wing conditions. 

The ageing per time unit was calculated sex-specifically by correlating the time intervals between capture and first recapture and the deterioration rate of wing condition. For Spearman rank correlation analyses, only days with at least five individuals per sex were considered, and their mean values were used. Furthermore, sex-specific behavioural patterns were investigated by using the X^2^-test. The influence of wind and cloud cover on the behaviour was also analysed with an X^2^-test. Potential sex-specific preferences of nectar plants were investigated with X^2^-test and X^2^-homogeneity test.

### 2.4. Population Demography

Based on the mark-release-recapture dataset, we conducted population modelling to assess population size as a potential indicator of habitat quality and to examine population structure for potential adaptations. The program Mark 8.2 and its module Popan [30], based on the Jolly–Seber method, was used to calculate the daily, sex-specific population size. Three parameters were calculated: ϕ (phi), the probability of survival; p, the probability of capture; and pent, the proportional recruitment. These parameters can be constant (.), sex-specific (g), time-factorial (t), linear (T) or quadratic (T^2^) and can have additive (g + t; g + T, …) or interactive relationships (g × t; g × T; …) [31]. Furthermore, the sampling effort (i.e., the time spent in the terrain) was considered as a covariant for the probability of capture. A saturated model consisting of these parameters was verified by a goodness-of-fit test with the program RELEASE. Based on this, various parameter combinations were calculated, and the best-supported model was determined using the corrected Akaike Information Criterion (AIC_C_) [32] and the lowest number of parameters [33].

### 2.5. Wing Condition

We applied linear and non-linear mixed-effects models to illustrate the ageing of the population based on the wing condition. These models didn’t converge with the female dataset. We then tested polynomial functions from the first to the 9th order to describe the ageing of the population and selected the best model based on the R^2^-value. Based on the breaking points for the different stages of ageing, we used linear models to describe these periods. We used the average wing wear of at least five specimens to avoid a sample size bias.

### 2.6. Mobility Parameters

We analysed movement patterns in the habitat to infer migration potential as well as resource use and availability. The collected GPS data of captures and recaptures were used to reconstruct movement patterns. These were imported into QGIS 3.8.3 [34], and the direct geographic distance between capture and first recapture was determined for each individual by creating a linear distance matrix layer with the vector analyses tool in the WGS 84 (EPSG4326) Coordinate Reference System. For assessing the minimum total distance moved, we summed up the distances between all points of capture. 

We performed a Shapiro–Wilk test to check for normal distribution by using the program R version 3.6.1 [35]. Since there was no normal distribution of the data (males p: 1.376 × 10^−10^, females p: 0.023), the test was followed by a Mann–Whitney U test to determine differences in distances travelled by males and females and by a two-sided Spearman’s rank correlation to analyse the influence of days since capturing on the distance. The travelled distances were divided into distance classes (20 m, 30 m and 50 m intervals) separately for each sex. The inverse cumulative percentage of these classes was determined, which corresponds to the probability density function, i.e., the dispersal kernel. To check for any potential artefacts caused by the chosen interval sizes, we analysed and compared three different intervals and size classes. 

Based on these classes, the probabilities of dispersal flights were investigated through distance extrapolation. Two frequently applied regression analyses were used: the negative exponential function (NEF) and the inverse power function (IPF). The NEF tends to underestimate rare long-distance movements, whereas the IPF may encounter problems with “zero” movements [36]. The data were linearly transformed with a semi-ln plot for the NEF analyses or with a double-ln plot for the IPF analyses. In both equations, P stands for the proportional probability that an individual will travel at least as far as the distance D, and a for the intercept of the regression. NEF works with the dispersal constant K as the slope, whereas IPF uses the variable n as the slope, which represents the effect of distance on dispersal [22].

(1) $$P_{NEF}=ae^{-kD}\;\mathrm{or}\;\ln P=\ln a-k\;D$$

(2) $$\mathrm{or}\;P_{IPF}=aD^{-n}\;\mathrm{or}\;\ln P=\ln a-n\left(\ln\;D\right)$$

We selected the best model and the most suitable interval size based on calculated stability indices R^2^ of the calculated curves, which corresponds to the proportion of explained variance of the dependent variable by the independent variable. This allowed extrapolations of the population’s proportion that should travel distances exceeding the extent of the study area. The calculations were performed separately for males and females.

## 3. Results

During 28 days with captures, 962 individuals (808 males; 154 females) were marked; 260 individuals (246 males; 14 females) were recaptured. This corresponds to a recapture rate of 27.0% (30.4% males, 9.1% females) and a sexratio of five males per female. We achieved up to five recaptures for males; females were recaptured not more than once.

### 3.1. Demography

The best-supported model with the lowest AIC_C_ value and the lowest number of parameters yielded an additive effect of sex and factorial time on the survival probability phi, an additive effect of sex and survey time on the capture probability p, and an interactive effect of sex and factorial time on the proportional recruitment pent and also on the number of individuals (Table 1). This model estimated population size of 2667 males, i.e., 580/ha (±37 SE). The population size of the females was estimated at 1110 individuals, i.e., 241/ha (±66 SE). The estimated sexratio was 2.4 males per female. Estimates based on the best supported Popan model were consistent with the actual daily recapture events.

**Figure 1:**
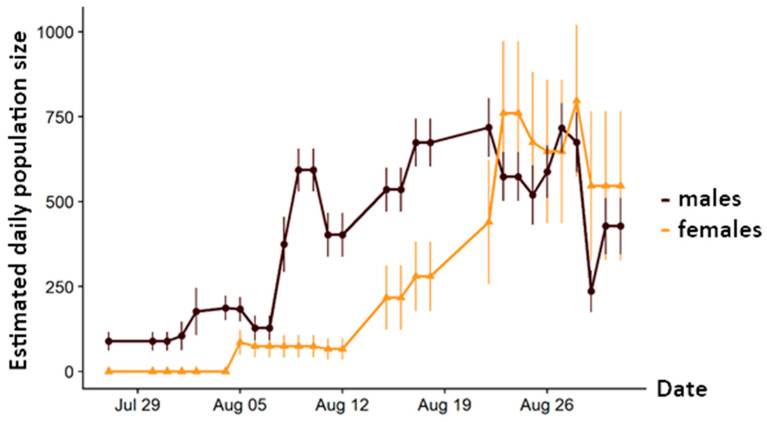
Estimated daily population size of both sexes of *Erebia pronoe* based on the best Popan model in MARK: Phi(g + t) p(g + hours) pent(g × t) N(g × t).

We observed distinct protandry. Thus, the first males were observed one week before the first females; they showed a strong increase from 7 August 2019 onwards (Figure 1). The first females were marked 5 August 2019; after two weeks of parallel development to males, they exceeded the daily numbers of males on 23 August 2019. The highest estimates were obtained for males and females on 27 and 28 August 2019, respectively.

**Figure 2:**
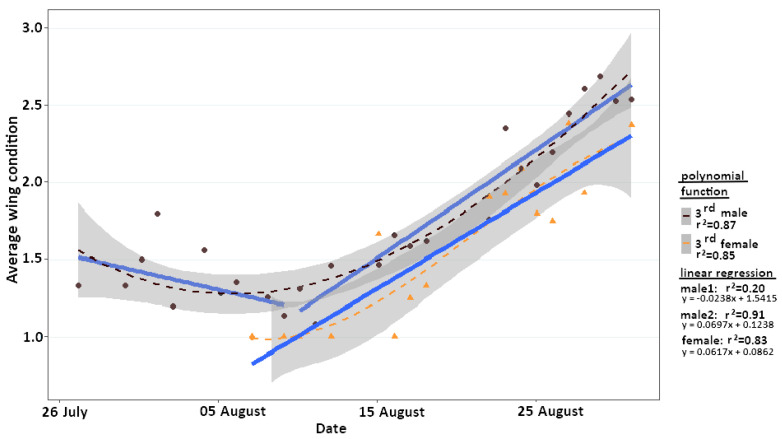
Changes in the average wing condition during the 2019 flight season of *Erebia pronoe* are categorised by sex. Males are indicated by brown circles, and females are indicated by orange triangles. Linear trends for both sexes are given and are based on the 3rd polynomial function. The 95% confidence interval of the polynomial function is given in grey.

We calculated changes in the average wing condition to assess the age structure of the population (Figure 2). Males, in general, had very good wing conditions for the first 16 days of the study and did not exhibit any deterioration until 11 August 2019 (y = −0.0238x + 1.5415; R^2^ = 0.205). Only from 12 August 2019 onwards, average wing condition deteriorated continuously (y = 0.0697x + 0.1238; R^2^ = 0.91) and significantly (*p* < 0.001). Due to later emergence and generally lower capture events, reliable data for females are only available for the second half of August. In this time slot, deterioration of females’ wing conditions (y = 0.0617x + 0.0862; R^2^ = 0.83) showed no significant difference from males (*p* = 0.69). Both sexes showed a highly significant correlation between the changes in wing condition and the time elapsed since first capture (Spearman rank correlation analysis: male rho = 0.93; female rho = 0.63; *p* < 0.001).

### 3.2. Mobility and Movement Patterns

**Figure 3:**
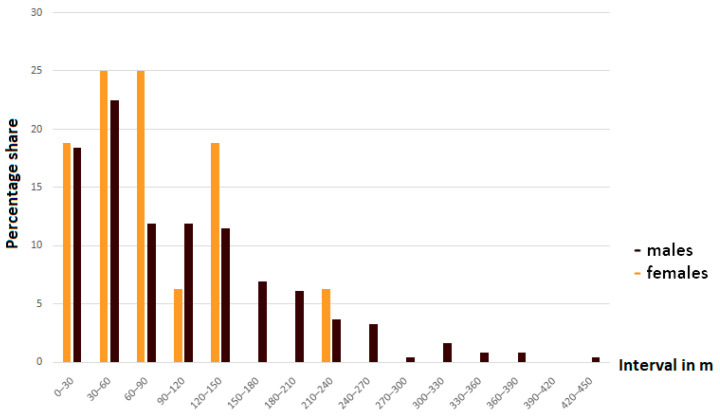
Percentage distribution of the sexes of *Erebia pronoe* over 30 m distance intervals corresponding to the distances travelled when first recaptured and divided by sex.

*Erebia pronoe* is a species with low mobility. Males (average dispersal distance: 106 m ± 86 SE; *n* = 245) tended to move somewhat further than females (71 m ± 58 SE; *n* = 16) but not significantly so (*p* = 0.15). Both sexes moved distances below 150 m in the majority of cases, but the percentage of males exceeding this distance (24.1%) was considerably higher than for females (6.2%) (Figure 3). The longest and shortest distances detected were 423 m and 3 m for males and 219 m and 8 m for females. 

We applied the NEF and IPF functions for extrapolating the potential for dispersal over longer distances. The highest stability indices (R^2^) for both functions to the inverse cumulative proportion values (based on distance classes) were obtained for 30 m intervals (Table 2). For both sexes, the fit of NEF was better than the fit of IPF. Following the NEF, dispersal of 1 km or more is unlikely (Table 3). Even if the NEF is prone to underestimate rare long-distance movements, the IPF results also give a low probability for these long-distance movements (i.e., males: 3 in 1000; females: 8 in 1000), which is consistent with our observations (Figure 3). 

Spearman’s rank correlation revealed a positive correlation of elapsed time between captures and migration distance. This effect was more pronounced in females (rho= 0.6679092, *p* = 0.003252, S = 185.97) than in males (rho = 0.1471955, *p* = 0.01128, S = 1964827).

### 3.3. Behavioural Differences between Sexes

Both sexes were observed with almost equal frequency in nectar uptake (Table 4). Flight activity was most frequently observed in males, whereas females were primarily encountered resting. Overall, both sexes differed highly significantly in their behaviour (χ^2^ = 131.1, df = 3, *p* < 0.001). The behaviour of males became more passive (*p* < 0.001) with increasing cloud cover and wind force, although this effect was only detectable for cloudiness of 70% or more and a wind force of level 3 (ES3) or more. 

### 3.4. Use of Nectar Plants

Members of the family Asteraceae were used as nectar sources in 77.3% of the cases. Most frequently visited were *Carlina acaulis* (37.0%), *Carduus defloratus* (18.4%) and *Leontodon hispidus* (13.2%). In addition to Asteraeceae, the family Dipsacaceae with *Scabiosa lucida* (12.3%) and the family Gentianaceae with *Gentianella campestris* (9.7%) were important. Sex-specific preferences were not detected (χ^2^ = 8.798, df = 8, *p* = 0.36).

## 4. Discussion

### 4.1. Population Size and Structure

Although we estimated a relatively high overall population density of 820 adult individuals per hectare, the proportion of female adults was comparatively low. This might be either caused by an actual low female population size or by biases associated with the methods. A biased sex ratio in the offspring, in emigration rates, and/or in survival rates of larvae/pupae can cause such distorted sex ratios in a population. None of these possible reasons mentioned above have so far been reported for such imbalanced sex ratios in the offspring of *Erebia*, and we have not observed such migration pattern either. The studied population displayed high levels of infection with the endoparasite *Wolbachia* (Wendt et al., unpublished), which is known to alter the sex ratio of populations in favour of the female offspring [37,38]. Higher mortality risk of the female pupae caused by the prolongated preimaginal phase is rather unlikely, considering the serial eclosion of both sexes. Therefore, we believe a bias due to methods as an explanation for the imbalanced population structure to be more likely. Studies that considered behavioural patterns found a correlation between population size and flight activity [22]. We assume that the rather low flight activity of female *E. pronoe* led to underrepresentation in marking and recapture events, which resulted in lower population estimates. Even in populations with a female bias, a higher capture probability of males can lead to a male over-representation in recapture studies [39]. It must also be considered that female behaviour and mobility are determined by numerous other factors and may not be constant. For example, factors such as larval plant availability can also lead to stronger, temporary changes in female mobility within a flight period [40]. Constant sex-specific influence may arise from landscape structures and topography. In studies on *Maculinea nausithous* and *Maculinea teleius*, strong barrier functions of topographic structures on female mobility have been demonstrated [41]. An inhibitory effect of the elevation ranges of our study area cannot be excluded and may have contributed to the low mobility of females and their underrepresentation. 

### 4.2. Adaptation to Extreme Conditions through Protandry and Serial Eclosion

The plateau phases in the population curve and the wing condition curve indicate the constant influx of freshly eclosed specimens into the population caused by serial eclosion. This serial eclosion is more visible in the case of males and also starts earlier compared to females. The earlier onset of male eclosion is considered beneficial for male reproductive success since it allows an immediate mating of freshly eclosed females, although, in high alpine areas, protandry can be a risk, especially for isolated populations. Extreme weather events can eradicate a major part of the population, hence causing a local collapse [42]. As a result, an earlier onset of the male flight period in combination with a prolonged phase of male eclosion provides the advantages of proterandry but also mitigates the risks of local extinction caused by weather caprices. Moreover, serial eclosion reduces the intraspecific pressure of eventual mass eclosion events and the hereon emerging need for emigration. Very similar population structures have also been witnessed in other high alpine butterflies (e.g., [22]) and are assumed to be an adaptation to high alpine environments. 

### 4.3. Resource Use

**Figure 4:**
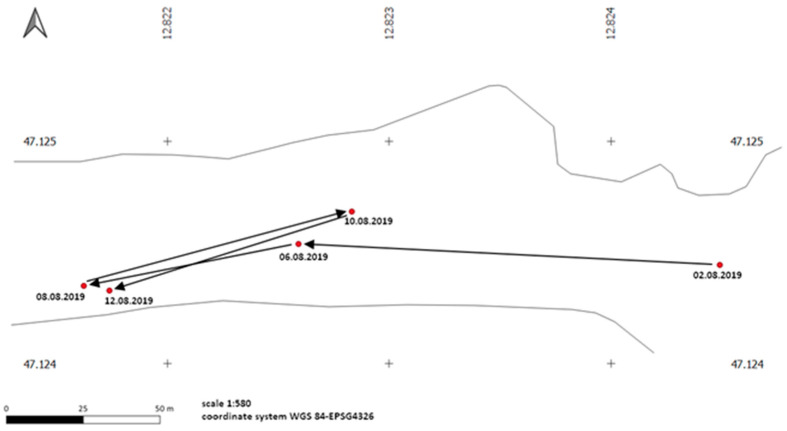
Example of movement patterns of a male of *Erebia pronoe* recaptured five times. The “+” symbols represent the grid of the coordinate system.

The analysis of the movement patterns of *Erebia pronoe* showed a rather sedentary behaviour and the formation of home ranges (Figure 4). These home ranges suggest a high habitat quality since the availability of resources determines the behaviour of butterflies [36,43], and apparently, the needs are fulfilled in small sub-areas of our study area. This is facilitated by the wide range of nectar plants used, which is consistent with Rapport’s extended rule [44]. From early/mid-July to mid/late August, the congeneric species *Erebia nivalis* is also common in our study area [22]. The larvae of *E. nivalis* have a biennial development but are also preferentially deposited on withered plant parts of *Festuca* species like *F. quadriflora*. Interestingly, despite the wide range of nectar plants used by *E. pronoe* and *E. nivalis*, no overlap was found. Both species use plant families that have very different flower morphology and colouration [22]. Therefore, it can be assumed that this is not a specific adaptation but a local, opportunistic use of resources. The wide range of larval food plants and nectar plants might mitigate the risk of phenological mismatch between hostplants and butterflies, a risk that has been observed several times in the context of climate change (e.g., [45]). We assume that the observed ranges of nectar plants used by *E. pronoe* rather reflect competitive mitigation than the actual foraging range of the species. The abundance of the genus *Festuca* in alpine habitats makes the competition for limited larval food plants unlikely.

### 4.4. Movement Patterns

We observed low mobility and movements of short distances but with significant differences between sexes. While males were significantly more active than females, they still moved mostly short distances and travelled larger distances only to establish home ranges. As a result, the average distances travelled between capture and first recapture did not increase as a function of time. Females, on the other hand, showed significantly lower mobility than males, but the distance travelled was positively correlated with time. Both of these behavioural traits can be explained by the population structure and resource use discussed above. The wide range of nectar plants and the commonness of the larval food plants do not force females to translocate larger distances due to the abundance of all resources needed. In addition, migration always imposes a risk and reduces the time for mating and oviposition [46]. Thus, reproductive success should initially be the highest in the patch of origin and exploitation of further areas beyond only increases this success when the female has already oviposited most of her eggs. Male harassment and the chance to lay fertilised eggs in other areas might explain the later increase in observed translocation as a function of time [47]. Due to the earlier eclosion of the males, they can establish a home range and patrol it in search of freshly eclosed females. This patrolling is reflected in the increased mobility and the low but constant migration performance of the males. The establishment of home ranges can reduce the effective population size [48] and further isolate a population in a fragmented landscape. Although detrimental on a population level, the avoidance of large-scale movements might reduce the risks posed by strong wind and bad weather in general and could be therefore beneficial on an individual level. The true migratory potential of the species may be significantly higher, as indicated by the observed maximum distances flown by the males. However, to answer this question, research in larger, potentially less resource-rich areas is required.

## 5. Conclusions

*Erebia pronoe* is a species that is well adapted to montane and alpine habitats because of the combination of moderate protandry and serial eclosion, the opportunistic use of resources as adults and its low mobility. The wide ecological niche reduces the risks of negative fitness consequences that could result from butterfly-host asynchrony. The serial eclosion of males reduces the risk of desynchronised adult emergence and the resulting risk for reproduction and stability of the population. At the same time, its relatively high abundance and stable populations make it a very good indicator species for the quality and the intactness of alpine rupicolous grasslands. It is likely that the female portion of the population is higher than predicted by our models, as previously discussed. Nonetheless, a higher population density of females would further support our statement regarding habitat quality. However, the population structure with serial eclosion and protandry is unaffected by the number of females. There might be an influence on the sedentary behaviour and migration potential during periods of resource scarcity, as may occur due to mass emergence and/or low habitat quality. However, the wide range of larval food plants and nectar plants should counteract intraspecific competition. We, therefore, assume that even a higher abundance of females would not affect the basic conclusions of our work.

## Figures and Tables

**Table 1:** Comparison of the best models of Popan 5.0 analyses for the estimates of the daily population size of *Erebia pronoe*: Akaike Information Criterion (AIC_C_) and number of parameters used, basic variables: Probability of survival (Phi), probability of recapture (P), proportional recruitment (Pent), the total number of individuals (N), dependent variables: sex (g); factorial (t), linear (T) and quadratic (T2) dependence of time; time invested in sampling per day (hours).

Model Nr.	Model	AIC_C_	Parameters
**1**	{Phi(g + t) p(g + hours) pent(g × t) N(g × t)}	2404.7462	34
**2**	{Phi(g × t) p(g × hours) pent(g + t) N(g × t)}	2408.9191	64
**3**	{Phi(g + t) p(g + hours) pent(g + t) N(g × t)}	2414.2151	18
**4**	{Phi(g + t) p(g × hours) pent(g × t) N(g × t)}	2416.0908	29
**5**	{Phi(g × t) p(g × hours) pent(g × t) N(g × t)}	2417.6189	30

**Table 2:** Stability index (R^2^) of the inverse power function (IPF) and the negative exponential function (NEF) based on calculations with 20, 30 and 50 m intervals of the covered distances of *Erebia pronoe*.

	20 m Intervals		30 m Intervals		50 m Intervals	
	IPF	NEF	IPF	NEF	IPF	NEF
Males	0.76	0.97	0.79	0.98	0.81	0.96
Females	0.79	0.94	0.83	0.93	0.80	0.93

**Table 3:** Percentage of *Erebia pronoe* individuals that were expected to disperse more than 1, 2, 3 or 5 km, calculated with inverse power function (IPF) and negative exponential function (NEF) based on 30 m intervals.

Distance	IPF Males	IPF Females	NEF Males	NEF Females
1 km	0.29	0.82	0.0003	3.50 × 10^−5^
2 km	0.07	0.29	4.27 × 10^−10^	6.50 × 10^−12^
3 km	0.03	0.15	5.86 × 10^−16^	1.21 × 10^−18^
5 km	0.01	0.07	1.10 × 10^−27^	4.15 × 10^−32^

**Table 4:** Percentage of individuals of *Erebia pronoe* in four different behavioural categories, divided by sex.

	Flying	Resting	Feeding	Mating
Males	57.7	33.2	8.9	0.2
Females	11.9	75.6	11.3	1.2

## Data Availability

The datasets used and/or analysed during the current study are available from the corresponding author on reasonable request.
